# Psychosocial Determinants of Quality of Life and Active Aging. A Structural Equation Model

**DOI:** 10.3390/ijerph17176023

**Published:** 2020-08-19

**Authors:** Laura Ponce de León, Jean Pierre Lévy Mangin, Soledad Ballesteros

**Affiliations:** 1 Department of Social Work, National Distance Education University (UNED), C/ Obispo Trejo, 2, 28040 Madrid, Spain; 2 Department of Administrative Sciences, Université du Québec en Outaouais (UQO), Bureau, B-2072, Pavillon Lucien-Brault, 101, rue Saint-Jean-Bosco, Gatineau, QC K1C 5J7, Canada; Jean-Pierre.Levy-Mangin@uqo.ca; 3 Department of Basic Psychology II, National Distance Education University (UNED), C/ Juan del Rosal, 10, 28040 Madrid, Spain; mballesteros@psi.uned.es

**Keywords:** active aging, memory, quality of life, social support, SEM

## Abstract

Population aging is the 21st century’s predominant demographic event. The old-age dependency ratio is projected to rise sharply in the next decades. Variables of health-related quality of life can be useful in designing interventions for promoting active aging to prevent dependency and save governments’ budgets. This study aims to find a model capable of explaining how psychosocial variables are related to improved quality of life during active aging, and if this relationship varies with age. Structural equation modelling (SEM) was used to examine the relationships among the availability of social resources, memory, depression, and perception of quality of life from three community senior centers in Madrid (Spain) in a sample of 128 older adult volunteers. The results suggest a psychosocial model where the availability of social support improves quality of life and explicit memory, reduces depression in active older adults, and where there are two main elements for understanding quality of life: perception of health and satisfaction. Importantly, age does not modify the interactions between variables, suggesting that their behavior is constant across aging. We concluded that the availability of social resources, understood not only as the people we interact with daily but also other family members, close friends, or institutions that could help in case of an emergency, allows people to avoid isolation and loneliness, increasing satisfaction and well-being in older adults. Professionals and policymakers should promote well-being by incorporating psychosocial variables related to personal satisfaction in the existential project, not only health, functional activity, or a friendly environment. Older adults need to feel that they are not alone, and in this sense, the availability of social resources is key.

## 1. Introduction

Due to the decline of fertility and the increase of longevity, most countries in the world are experiencing a significant growth in the number of older adults. It is expected that this number will double in the next 30 years, reaching 1.5 billion by 2050. The number of people aged 65 or over is expected to increase from 6% today to 16% by 2050. Globally, [1] a person aged 65 years in 2015–2020 is expected to live, on average, an additional 17 years; by 2045–2050 this will have increased to 19 years. Most older people reside in Asia, Europe and North America.

Japan, Finland, Sweden, Greece, Italy, Germany and Spain are among the countries in the world with a larger number of older adults. In 2060 [2] forecasts place China at first place with 44.3% of its population over 60, followed by Japan with 42.5%, Korea with 41.5%, Spain with 41.5%, Portugal with 41.2%, Greece with 40.8%, and Italy with 40.7%.

Data from the National Statistics Institute indicated that in January 2019 the population in Spain was 47,026,200 persons. People over 65 were approximately 19.3%, and almost 6% were over 80 years old [3]; furthermore, these data showed that almost 31% of the population over 65 years of age was over 80 years old, evidence of so-called aging of aging.

With the huge increase of life expectancy in the European countries as well as in other developed countries, new needs for long-term care arise in order to guarantee quality of life for an increasing older adult population [4]. Some of those needs might be fulfilled by the family, but others should be covered by public institutions. Both families and society in general will face greater pressure for financial aid. Active aging could be a solution to this problem. It is a new paradigm associated with the classic gerontology theories: activity theory, continuity theory or successful aging [5], and other terms such as “productive aging”, “aging well”, “senior wellness” or “living well” [6].

Almost two decades ago, the Second World Assembly on Aging defined active aging as the way of improving opportunities for participation, security, and health in order to enrich quality of life as people age. It permits the elderly to understand their potential for physical, social, and mental prosperity in their lifetime and to take part in society as indicated by their necessities, wants, and limits. The determinants of active aging are related to social service systems and health, physical environment, personal factors, behaviors, social relationships, and economy [7]. Later, the United Nations Economic Commission for Europe [8] proposed the Active Aging Index (AAI) that includes 22 indicators grouped into four domains: 1. Participation in society; 2. Capacity and enabling environment for active aging; 3. Independent, healthy and secure living; and 4. Employment. In the analytical report on AAI published in 2019 [9], the conclusions and challenges for the future to promote active aging were: focus on inequalities (lower education and income); paying attention to rural and urban differences; identifying life course events and trajectories; accessibility and affordability of health, social, and long-term care services; and considering the diversity of individual and cultural perspectives. It is important to have a multidimensional view to promote active aging.

According to these conclusions, elements of quality of life and active aging are associated with biopsychosocial components. Promoting active aging and preventing dependency are key to saving budgets. To reach this objective, it is necessary to understand what variables are essential to improve quality of life in active aging.

Many are the components of healthy aging, including physical and psychological health, independence, finances, the practice of leisure activities, mental activity, social contacts, community services, and support [10]. Importantly, a meta-analysis conducted with almost 300 studies described how social support is highly connected to quality of life, both in the number of significant others existing, the quality of the relationships, and the support system [11]. In this way, there are three main factors: physical health, social support, and psychological well-being [12].

Overall multidimensional models of active aging make use of distinct elements. For example, quality of life, cognition, mental health, and self-assessed physical health compose the meaning of this concept [13]. Other studies consider factors such as physical health, social support, psychological health, and the practice of leisure activities [14]. Other references focused on aging-friendly environments (cities or communities), and suggest three domains of active aging: health, security, and participation [5].

Our model attempts to clarify the relationship among psychosocial factors. Researchers have studied many variables and different outcomes have been obtained depending on the type of studies. There is a large number of studies linking active aging and quality of life. The length of this article prevents us from referring to all of them, so we will select those that can theoretically justify the relationships between the variables chosen in our study.

Loneliness and daily forgetfulness are two of the major concerns of active older adults and are related to each other. Many studies suggest that affective and depressive disorders have a negative impact on quality of life [15,16]. Social interaction and support are related to life satisfaction, and to the physical and mental well-being of older people [17,18,19,20]. Appropriate social relationships could determine personal success associated with happiness and quality of life [21]. Speaking frequently with friends could reduce the prevalence of depressive symptoms [22] and doing exercise and participating in recreational activities with friends or family members could improve mental health and happiness [23]. For daily forgetfulness, participation in a memory program could be an effective recommendation, not only to improve memory but also for changing perceived quality of life [24]; therefore, memory could be a good indicator of well-being. We considered all these connections in the structure of the current model.

Based on our previous work [25], we suggest another set of connections to clarify the role of the psychosocial factors during active aging. These are: (1) Depression and social resources are correlated negatively between them and quality of life; (2) explicit memory is a direct detector of the perception of quality of life; (3) depression negatively influenced the perception of quality of life; (4) availability of social resources positively influenced perception of quality of life and improved memory; (5) view of personal quality of life could be explained by health perception and life satisfaction; (6) age could change the psychosocial connections during active aging.

## 2. Methods

### 2.1. Participants

In this study 128 older adults were involved from three senior community centers in Madrid (Spain) located in three different geographical areas (Chamberí, Fuencarral, and Tetúan) with different socioeconomic levels, which allowed us to control effects of this variable in the results. In this sense, the social workers at the centers helped us to select the sample because they know all social cases and they provided us a list based on the inclusion criteria of our study.

The average age was 75.24 years (SD = 5.93), and members had finished an average of five years of essential education. Fifteen percent of participants were men (22), and 85 percent were women (106). The inclusion criteria were: (1) active aging and independent living, (2) basic school studies, (3) to be retired and be over 60 years old, (4) Geriatric Depression Scale (GDS) score between 0 and 9, and (5) Mini-Mental State Examination (MMSE) score equal to or greater than 27. Exclusion criteria were: (1) hearing or visual impairment, (2) diagnosis of mental disease, (3) participation in an active psychosocial training program.

Previous to participation in the study, older volunteers signed a detailed consent form. The study was authorized by the Ethics Committee of the National Distance Education University, in accordance with the Spanish Universities Declaration adopted in the Second Meeting of Bioethics Committees and with the ethical standards laid down in the Helsinki Declaration. This study is enrolled within the doctorate program “Aging and Neurodegenerative Diseases.” The management of the Regional Welfare Service of the Community of Madrid also approved this study.

### 2.2. Latent Variables. Description of Tasks Related

Explicit Memory: The Wechsler Memory Scale—Third Edition [26] was used to measure memory, using two subtests of the scale: the Delayed Visual Index and the Delayed Auditory Index.

Depression: To assess depression, we used the reduced version of the GDS [27] with 15 items. In this study we considered the range of scores between 0 to 9, that indicates no clinical depression.

Social Resources: Social Resources Scale (OARS) [28] was used to measure subjective satisfaction with relationships and objective data (frequency and quantity of social contacts and instrumental support).

Perceived Quality of Life: We used the Philadelphia Scale of Satisfaction [29] which measures three scopes: disturbance, disposition toward aging, and disappointment with isolation.

Nottingham Health Profile (NHP), Spanish version [30]: The Nottingham Health Profile is proposed for essential medical services, to give a concise sign of a patient’s apparent enthusiasm, social, and physical health problems. A lower score on this scale means greater health perception.

These assessment instruments were selected because they were simple and fast, so as not to exceed one hour in the interview. All tests selected passed the criteria of validity and reliability. From the Wechsler Memory Scale, we selected just two indexes because delayed memory activities are the most important to explain explicit memory.

### 2.3. Procedure

Participants who passed screening tests (GDS and MMSE) were tested individually in a counterbalanced test order in a room of the community center.

### 2.4. Structural Analysis

We applied structural equation modelling (SEM), especially the AMOS 16 (Analysis of Moment Structures) program, to investigate the relationships among the psychological variables in a sample of 128 older volunteers. This program works under the SPSS platform (Statistical Package for the Social Sciences) and uses the structural equation model to confirm and explain the conceptual models that deal with attitudes, perceptions, and other factors that determine the behavior of the variables. There were no missing data. In this sense, we considered three indices: χ^2^, the root-mean-square error of approximation (RMSEA), and the comparative fit index (CFI). Good model fit was accepted when CFI was more than 0.90 and RMSEA values were near zero. The structural causality of the interactions between the variables allows a logical and rational approach to the theory without independent variable manipulation. Phenomena, such as active aging, could be explained using the observed variables from the psychological scales to construct the graphic model.

## 3. Results

**Figure 1:**
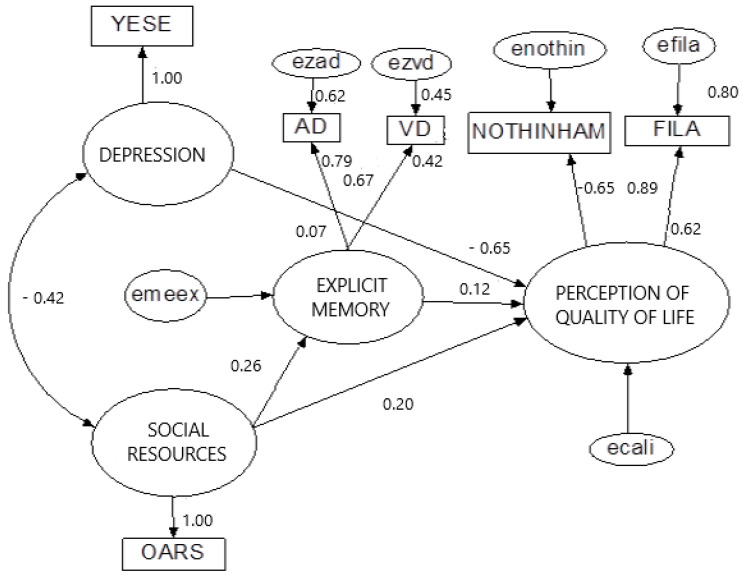
Active aging model. Relationship of social resources, depression, explicit memory and perception of quality of life. Source: Self-made. Note: Main goodness-of-fit indices in the active aging model: χ^2^(6, *N* = 128) = 7.708, *p* = 0.9260; normal fit index = 0.992; comparative fit index (CFI) = 1.00; root-mean-square error of approximation (RMSEA) = 0.00; goodness of fit = 1.00; adjusted goodness of fit = 0.997; ezvd = Error; ezad = Error; VD = delayed visual index; AD = delayed auditory index; YESE = Yesavage’s Geriatric Depression Scale (Yesavage, 1986); FILA = Philadelphia Scale of Satisfaction; OARS = Social Resources Scale; ecali = Error; NOTHINHAM= Nottingham Health Profile (NHP); enothin = Error. Estimate, SE, CR, P, Label data and Model Fit Summary are available in Supplementary Materials.

On the basis of our theoretical predictions, we obtained a definitive model that could explain active aging with a psychosocial perspective (see Figure 1).

The active aging model appears with four latent variables with six indicators and six degrees of freedom. We tested unidimensionality with Cronbach’s alpha (reliability index). The psychosocial scales and memory subtests all followed validity and reliability criteria (see Table 1).

The model shows extracted variance and reliability indices in latent dimensions (see Figure 1). Requirements for acceptance this model as a valid confirmatory model of the empirical data were all fulfilled. Good mathematical fit happens when RMSEA is equal to 0 and CFI is 1.

Our structural model is composed of three independent latent variables (social resources, explicit memory, and depression) and one level of dependency (quality of life). The two independent latent variables (social resources and depression) were directly correlated. Principal effects were (see Figure 1):

1. The accessibility of social resources varied negatively with depression (−0.42) and positively with explicit memory (0.26). A person with good availability of social resources could reduce depression and improve explicit memory.
2. The latent variables of social resources, depression, and explicit memory directly influenced the perception of quality of life, with a negative value for depression (−0.65) and a positive value for social resources (0.20) and explicit memory (0.12).
3. The latent variable perception of quality of life is represented by two objective variables which mean “feel satisfaction in life” (0.89) and “have a good perception about health” (−0.65) (note that a lower score in the Nottingham Health Profile implies a better perception of health, due to the negative estimate value).

Participants were divided into two groups to investigate the effect of age in the model (young-old 60–75 years, and older-old 76–95 years). Age had no effect in the model because causal invariance results were not significant because estimates were similar.

According to the hypotheses formulated in the introduction, we could confirm the first, namely, the inverse relationship between affective state (depression) and social resources to explain quality of life was (−0.42). On the other hand, the second hypothesis was rejected because explicit memory did not have a direct effect on perceived quality of life, with a low value (0.12). The rest of the hypotheses were confirmed. Thus, depression negatively influenced the perception of quality of life (−0.65), availability of social resources positively influenced the perception of quality of life (0.20) and improved memory (0.26), and perception of quality of life could be explained by perception of health (−0.65) and satisfaction (0.89). The last hypothesis was rejected because age had no effect in the model. Estimate values were very similar.

## 4. Discussion

In the present study, we used SEM to investigate the relationships among memory, the availability of social resources, depression, and the perception of quality of life in a sample of older adult volunteers from several community senior centers in Madrid (Spain). Many studies have examined psychosocial variables as a way to try to understand quality of life. It is known that a positive association of quality of life is observed when individuals have wealth, higher education, and strong social capital components such as being currently married, social activity, civic engagement, trust, solidarity, and enjoying robust psychological resources [31].

There are only a few articles that relate quality of life with explicit memory using SEM [25]. We used two measures from the Wechsler Memory Scale, the Delayed Visual Index (VD) and the Delayed Auditory Index (AD) because both could explain cognitive decline. Explicit memory is necessary for the performance of activities of daily life. When this type of memory declines, the quality of life of older adults is low. Our model explains that explicit memory is not a direct signal of perception of quality of life, although everyday forgetfulness is [32]. For future studies, we will design a new model taking into account a subjective perception of daily forgetfulness together with objective memory measures. In a previous SEM study [33], social activity directly affected subjective memory complaints (−0.31) and depression (−0.28). In the present study, social resources directly affect explicit memory (0.26) and depression (−0.42). This could be a way to improve estimates.

As indicated by our graphic model, maximum memory execution was accomplished when the accessibility of social resources improved [34], maybe because social interactions are a way to put memory to work, avoiding cognitive decline [35]. Longitudinal studies suggest that participation in social activities could be a prevention factor to avoid cognitive decline [36].

The most important result of the current research is the availability of social resources to enhance mood, memory, and perception of quality of life (satisfaction and view of well-being) [18,37,38]. According to Malone and colleagues, psychosocial commitment in mid-adulthood has advantages for later-life cognitive and emotional health [39]. Our study suggests that mental health problems (affective disorder) were negatively connected to healthy aging. Individuals who feel blue lose personal satisfaction, and consequently, quality of life [13]. This discussion is in accordance with the negative relationship between depression and perception of quality of life (−0.65) obtained in our model.

Following the results of the graphic model presented in Figure 1, social ecological models in health and integral coordination equipment are essential in active aging. Another SEM study used other quality of life dimensions, including home conditions, health autonomy, social support, family, and social activities [40]. In this sense, social workers, educators, psychologists, doctors, and nurses should manage social resources to offer the elderly the feeling that they are not alone.

The connection between community well-being and lifelong learning is proposed from a social network perspective that advances an active and engaged lifestyle [41]. In Taiwan, senior learners who participated in learning group activities expressed positive perceptions of quality of life and satisfaction [21]. In fact, social workers should enrich social environments with this kind of social activity, such as painting, reading, yoga, sports, travel, learning English, information, leisure activities, and so on. Older adults who participated in socio-educational activities at social community centers had more satisfaction and associationism [42]. Moreover, social workers should inform older adults about available social welfare services (domiciliary services, day services, residential services, assistance technology, etc.). They should also use mediation to improve contacts with family members [43]. Professionals and policymakers should advance well-being by incorporating psychosocial variables related with personal satisfaction in the existential project of older adults, not only considering health or functional activity [12].

Taking into account ecological perspectives, many studies suggest that age-friendly communities (characterized by social participation, secure living, social connections, and lifelong learning) have positive effects on self-perceived health [44] and life satisfaction [45]. We obtained the same result in our model. Having good social resources available improves quality of life (satisfaction and perception of health). Active aging strategies to improve quality of life could include not staying at home, interactionism to avoid isolation, social participation (volunteerism and participation activities), active attitudes and learning, physical activity, and management of daily life at home [46].

In this study, social resources analyzed using the OARS scale offer a reduced view of this variable: only interactions and people or institutions available to help in case of urgency. In future studies, it would be appropriate to use other scales or questionnaires that allow an assessment of the participation of older people in society, how they perceive safety, and [47] the advantages of learning throughout life: three interesting elements that could improve our latent variable perception of social resources.

Our model shows a good fit index. However, we could improve these data by applying the same scale for each variable and expanding the sample. SEM requires a large sample to stabilize the average (our sample was formed by 128 older volunteers). Roughly 200 participants would be suggested [48]. The maximum likelihood optimization method is used to offer greater consistency when the sample is huge. We follow the rule of stability of the average that suggests 10 data points for each variable observed [49].

## 5. Conclusions

The present study suggests that a main element which must be considered to improve the quality of life of older adults is to provide them with optimal social resources, not just a person to speak to or to interact with, but also people that could help them in case of an emergency. In this sense, having specialized social services available for meeting their needs would be necessary too. Families and friends should be the first option, but what happens if these fails? If they fail, older adults could be sad or have the blues. To avoid this negative situation, they should feel the support of someone (a professional) or something (a service) to improve their mood, life satisfaction, or their perception of health and well-being. Furthermore, this trend does not vary with age; older adults want to feel accompanied and supported throughout their life cycle. The professionals of reference of social service are social workers, who could play a relevant role monitoring family cases, designing activities or programs, and managing social services.

The ecological perspective and the study of the environment is essential to guarantee participation, security, and learning throughout life. Policymakers should be oriented to enhancing age-friendly communities by promoting education, income, social cohesion, solidarity, volunteering, participating in social groups, political participation, social connections, lifelong learning, services, transport, leisure, etc.

For all these reasons, developing professional interventions focused on these psychosocial variables, mainly the perception of available social resources during active aging, would be key to preserving well-being, preventing dependency, and saving budgets. This article proposes a model to explain how to promote successful aging from a psychosocial perspective. This information could be useful for designing professional programs directed to older adults.

## Figures and Tables

**Table 1:** Extracted variance and reliability.

Variables in Model		
Latent Variable	Reliability	Extracted Variance
Depression	1	1
Explicit memory	0.71	0.53
Perceived quality of life	0.75	0.60
Social resources	1	1

Source: Self-made.

## Supplementary Materials

The following are available online at https://www.mdpi.com/1660-4601/17/17/6023/s1, Table S1: Estimate, SE, CR, P, Label data and Model Fit Summary.
