# Supplementary material for: Psychosocial Determinants of Quality of Life and Active Aging. A Structural Equation Model
Source: Int J Environ Res Public Health. 2020 Aug 19;17(17):6023. doi: 10.3390/ijerph17176023 (PMC7503318; doi:10.3390/ijerph17176023)
Supplement: Supplementary file 1 [file ijerph-17-06023-s001.pdf]

**ANNEXES**

***Estimates (global - Default model)***

***Scalar Estimates (global - Default model)***

***Maximum Likelihood Estimates***

***Regression Weights: (global - Default model)***

| | | | Estimate | S.E. | C.R. | P | Label |
|---|---|---|---|---|---|---|---|
| MEMORY_EXPLICIT | <--- | RESOURCES_SOCIAL | 2,681 | 1,114 | 2,407 | ,016 | |
| PERCEPTION_QUALITY_ DE LIFE | <--- | RESOURCES_SOCIAL | ,523 | ,204 | 2,566 | ,010 | |
| PERCEPTION_QUALITY_ DE LIFE | <--- | DEPRESSION | -,883 | ,104 | -8,529 | *** | |
| PERCEPTION_QUALITY_ DE LIFE | <--- | MEMORY_EXPLICIT | ,031 | ,023 | 1,330 | ,184 | |
| OARS | <--- | RESOURCES_SOCIAL | 1,000 | | | | |
| YESE | <--- | DEPRESSION | 1,000 | | | | |
| AD | <--- | MEMORY_EXPLICIT | 1,000 | | | | |
| VD | <--- | MEMORY_EXPLICIT | ,848 | ,342 | 2,481 | ,013 | |
| FILA | <--- | PERCEPTION_QUALITY_ OF LIFE | 1,000 | | | | |
| NOTHINHAM | <--- | PERCEPTION_QUALITY_ OF LIFE | -3,084 | ,445 | -6,926 | *** | |

***Standardized Regression Weights: (global - Default model)***

| | | | Estimate |
|---|---|---|---|
| MEMORY_EXPLICIT | <--- | RESOURCES_SOCIAL | ,261 |
| PERCEPTION_QUALITY_ DE LIFE | <--- | RESOURCES_SOCIAL | ,202 |
| PERCEPTION_QUALITY_ DE LIFE | <--- | DEPRESSION | -,654 |
| PERCEPTION_QUALITY_ DE LIFE | <--- | MEMORY_EXPLICIT | ,122 |
| OARS | <--- | RESOURCES_SOCIAL | 1,000 |
| YESE | <--- | DEPRESSION | 1,000 |
| AD | <--- | MEMORY_EXPLICIT | ,789 |
| VD | <--- | MEMORY_EXPLICIT | ,669 |
| FILA | <--- | PERCEPTION_QUALITY_ OF LIFE | ,893 |
| NOTHINHAM | <--- | PERCEPTION_QUALITY_ OF LIFE | -,647 |

***Covariances: (global - Default model)***

| | | | Estimate | S.E. | C.R. | P | Label |
|---|---|---|---|---|---|---|---|
| DEPRESSION | <--> | RESOURCES_SOCIAL | -1,211 | ,280 | -4,320 | *** | |

***Correlations: (global - Default model)***

| | | | Estimate |
|---|---|---|---|
| DEPRESSION | <--> | RESOURCES_SOCIAL | -,415 |

***Variances: (global - Default model)***

| | Estimate | S.E. | C.R. | P | Label |
|---|---|---|---|---|---|
| DEPRESSION | 5,580 | ,700 | 7,969 | *** | |
| RESOURCES_SOCIAL | 1,526 | ,191 | 7,969 | *** | |
| emeex | 150,251 | 66,854 | 2,247 | ,025 | |
| ecali | 3,834 | 1,161 | 3,303 | *** | |
| ezad | 97,863 | 64,409 | 1,519 | ,129 | |
| ezvd | 143,406 | 48,902 | 2,933 | ,003 | |
| efila | 2,581 | 1,102 | 2,343 | ,019 | |
| enothin | 134,090 | 19,583 | 6,847 | *** | |

***Squared Multiple Correlations: (global - Default model)***

| | Estimate |
|---|---|
| MEMORY_EXPLICIT | ,068 |
| PERCEPTION_QUALITY_ OF LIFE | ,623 |
| NOTHINHAM | ,419 |
| FILA | ,798 |
| VD | ,447 |
| AD | ,622 |

***Model Fit Summary***

***CMIN***

| Model | NPAR | CMIN | DF | P | CMIN/DF |
|---|---|---|---|---|---|
| Default model | 15 | 7,708 | 6 | ,260 | 1,285 |
| Saturated model | 21 | ,000 | 0 | | |
| Independence model | 6 | 221,651 | 15 | ,000 | 14,777 |

***RMR, GFI***

| Model | RMR | GFI | AGFI | PGFI |
|---|---|---|---|---|
| Default model | 3,701 | ,981 | ,933 | ,280 |
| Saturated model | ,000 | 1,000 | | |
| Independence model | 33,456 | ,606 | ,449 | ,433 |

***Baseline Comparisons***

| Model | NFI Delta1 | RFI rho1 | IFI Delta2 | TLI rho2 | CFI |
|---|---|---|---|---|---|
| Default model | ,965 | ,913 | ,992 | ,979 | ,992 |
| Saturated model | 1,000 | | 1,000 | | 1,000 |
| Independence model | ,000 | ,000 | ,000 | ,000 | ,000 |

***Parsimony-Adjusted Measures***

| Model | PRATIO | PNFI | PCFI |
|---|---|---|---|
| Default model | ,400 | ,386 | ,397 |
| Saturated model | ,000 | ,000 | ,000 |
| Independence model | 1,000 | ,000 | ,000 |

***NCP***

| Model | NCP | LO 90 | HI 90 |
|---|---|---|---|
| Default model | 1,708 | ,000 | 13,114 |
| Saturated model | ,000 | ,000 | ,000 |
| Independence model | 206,651 | 162,176 | 258,571 |

***FMIN***

| Model | FMIN | F0 | LO 90 | HI 90 |
|---|---|---|---|---|
| Default model | ,061 | ,013 | ,000 | ,103 |
| Saturated model | ,000 | ,000 | ,000 | ,000 |
| Independence model | 1,745 | 1,627 | 1,277 | 2,036 |

***RMSEA***

| Model | RMSEA | LO 90 | HI 90 | PCLOSE |
|---|---|---|---|---|
| Default model | ,047 | ,000 | ,131 | ,445 |
| Independence model | ,329 | ,292 | ,368 | ,000 |

***AIC***

| Model | AIC | BCC | BIC | CAIC |
|---|---|---|---|---|
| Default model | 37,708 | 39,458 | 80,488 | 95,488 |
| Saturated model | 42,000 | 44,450 | 101,893 | 122,893 |
| Independence model | 233,651 | 234,351 | 250,763 | 256,763 |

***ECVI***

| Model | ECVI | LO 90 | HI 90 | MECVI |
|---|---|---|---|---|
| Default model | ,297 | ,283 | ,387 | ,311 |
| Saturated model | ,331 | ,331 | ,331 | ,350 |
| Independence model | 1,840 | 1,490 | 2,249 | 1,845 |

***HOELTER***

| Model | HOELTER .05 | HOELTER .01 |
|---|---|---|
| Default model | 208 | 278 |

| Model | HOELTER .05 | HOELTER .01 |
|---|---|---|
| Independence model | 15 | 18 |
